# Massive Pericardial Effusion as a Manifestation of Severe Autoimmune Hypothyroidism in an Adolescent With Down Syndrome: Importance of Early Recognition and Thyroid Hormone Replacement

**DOI:** 10.1002/ccr3.73795

**Published:** 2026-10-24

**Authors:** Bivek Mishra, Pujan Pokharel, Abinash Karki, Oshin Rimal, Ayush Bakhrel, Diwakar Koirala, Ramesh Sapkota

**Affiliations:** ^1^ Department of General Practice and Emergency Medicine B.P Koirala Institute of Health Sciences Dharan Nepal; ^2^ Department of Cardiology B.P Koirala Institute of Health Sciences Dharan Nepal; ^3^ Birat Medical College and Teaching Hospital Tankisinuwari Nepal; ^4^ Department Internal Medicine B.P Koirala Institute of Health Sciences Dharan Nepal; ^5^ B.P Koirala Institute of Health Sciences Dharan Nepal

**Keywords:** Down syndrome, hypothyroidism, pericardial effusion, thyroid function screening

## Abstract

The clinical features of severe hypothyroidism may overlap with those of Down syndrome, delaying diagnosis until complications such as myxedema crisis and massive pericardial effusion develop. Regular thyroid function screening is essential, and hypothyroidism‐associated massive pericardial effusion can often be treated conservatively with levothyroxine in the absence of cardiac tamponade.

## 1 Introduction

Individuals with Down syndrome are at increased risk of developing hypothyroidism throughout childhood and adolescence [1]. Myxedema crisis (decompensated hypothyroidism) is a rare but life‐threatening complication of severe hypothyroidism characterized by progressive slowing of physical and mental activity, hypothermia, bradycardia, hypotension, hypoventilation, and altered mental status, which may progress to multiorgan dysfunction if left untreated [2]. Myxedema crisis is well recognized in adults, but it is extremely rare in the pediatric population [3].

Severe hypothyroidism may also lead to pericardial effusion. It occurs in 10%–30% of adults with hypothyroidism but is rare in children and adolescents. Most pediatric cases reported in the literature have been described in patients with Down syndrome (Table 3) [12]. Available literature suggests that even massive pericardial effusions associated with hypothyroidism in these patients may resolve following thyroid hormone replacement alone [8, 11].

Recognition of severe hypothyroidism may be particularly challenging in this population. Several manifestations of hypothyroidism, including developmental delay, reduced cognitive function, decreased activity, constipation, hypotonia, and behavioral changes may overlap with features of Down syndrome, leading to delayed diagnosis and treatment. For this reason, The American Academy of Pediatrics recommends verification of newborn thyroid screening results, followed by TSH testing at 6 months, 12 months, and annually thereafter in children with Down syndrome [13]. Similar screening recommendations are not uniformly implemented in many low and middle‐income countries like Nepal, and this may lead to delayed diagnosis of hypothyroidism in resource‐limited settings.

We report a teenager with Down syndrome who presented with respiratory distress and features of myxedema crisis secondary to severe autoimmune hypothyroidism which was further complicated by sepsis and bilateral community acquired pneumonia, and massive pleural and pericardial effusions.

## 2 Case Presentation

A 16‐year‐old male with Down syndrome presented to the emergency department with progressive generalized body swelling and shortness of breath.

According to his mother, the patient had been experiencing gradual swelling of the face and lower limbs for nearly one and a half months, which progressively involved the entire body. During the same period, he became increasingly lethargic, less interactive, and more irritable than usual. He also had a history of intermittent constipation. There was no history of headache, neck pain, vision problems, or head trauma. The symptoms progressed slowly and the underlying condition remained undiagnosed during this period. Four to five days before presentation, he developed shortness of breath, which worsened significantly over the last 2 days. There was no history of chest pain, palpitations, abnormal body movements, or loss of consciousness.

His only known medical condition was Down syndrome.

On presentation, the patient appeared ill and drowsy. His pulse rate was 88 beats/min; blood pressure was 90/60 mmHg; respiratory rate was 26 breaths/min with an irregular breathing pattern and use of accessory muscles; and oxygen saturation was 78% on room air. The jugular venous pressure was not elevated. On physical examination he had a typical appearance of Down syndrome with a flat nasal bridge, small low‐set ears, short neck, and a single transverse palmar (simian) crease. He had generalized non‐pitting edema with facial puffiness and gross abdominal distension. Respiratory system examination revealed bilateral basal crepitations with markedly decreased air entry over the left side. On cardiac examination the cardiac apical impulse was not visible. The apex beat was not felt. On auscultation, the heart sounds were distant. He had no murmur or pericardial rub. Neurological assessment showed decreased mental status; he was drowsy but arousable and intermittently irritable during examination.

Baseline investigations showed mild anemia (Hb 10.5 g/dL), leukocytosis (TLC 14,930 cells/mm^3^) with neutrophilic predominance, and thrombocytopenia (platelet count 100,100 cells/mm^3^). Renal function tests showed blood urea of 41.95 mg/dL and serum creatinine of 1.46 mg/dL. Liver function tests revealed elevated transaminitis (AST 243.38 U/L and ALT 228.81 U/L) along with raised alkaline phosphatase levels (347.39 U/L). Urinalysis showed 2+ proteinuria, while the remaining parameters were within normal limits.

Chest radiography revealed a massive left‐sided pleural effusion with a small right‐sided pleural effusion and an enlarged cardiac silhouette as shown in (Figure 1). Pleural fluid analysis revealed an exudative effusion with protein 3.91 g/dL, LDH 132.48 U/L, glucose 82.06 mg/dL, ADA 2.92 U/L, and total leukocyte count of 2200 cells/mm^3^ with granulocyte predominance. The patient was initially diagnosed as pneumonia and sepsis. Discovery of cardiomegaly on chest X‐ray prompted an echocardiogram which demonstrated a large pericardial effusion shown in (Figure 2) without evidence of tamponade physiology. A limited diagnostic pericardiocentesis was performed, and approximately 20 mL of pericardial fluid was aspirated for diagnostic evaluation. Pericardial fluid analysis showed lymphocyte predominance with a total leukocyte count of 10 cells/mm^3^ (95% lymphocytes, 5% granulocytes), protein concentration of 4.96 g/dL, albumin 5.72 g/dL, LDH 266.47 U/L, and RBC count of 79.94 cells/μL. Gram stain and bacterial culture were negative. Bedside point‐of‐care ultrasonography (POCUS) additionally revealed incidental findings of multiple gallstones and right sided nephrolithiasis.

**FIGURE 1:**
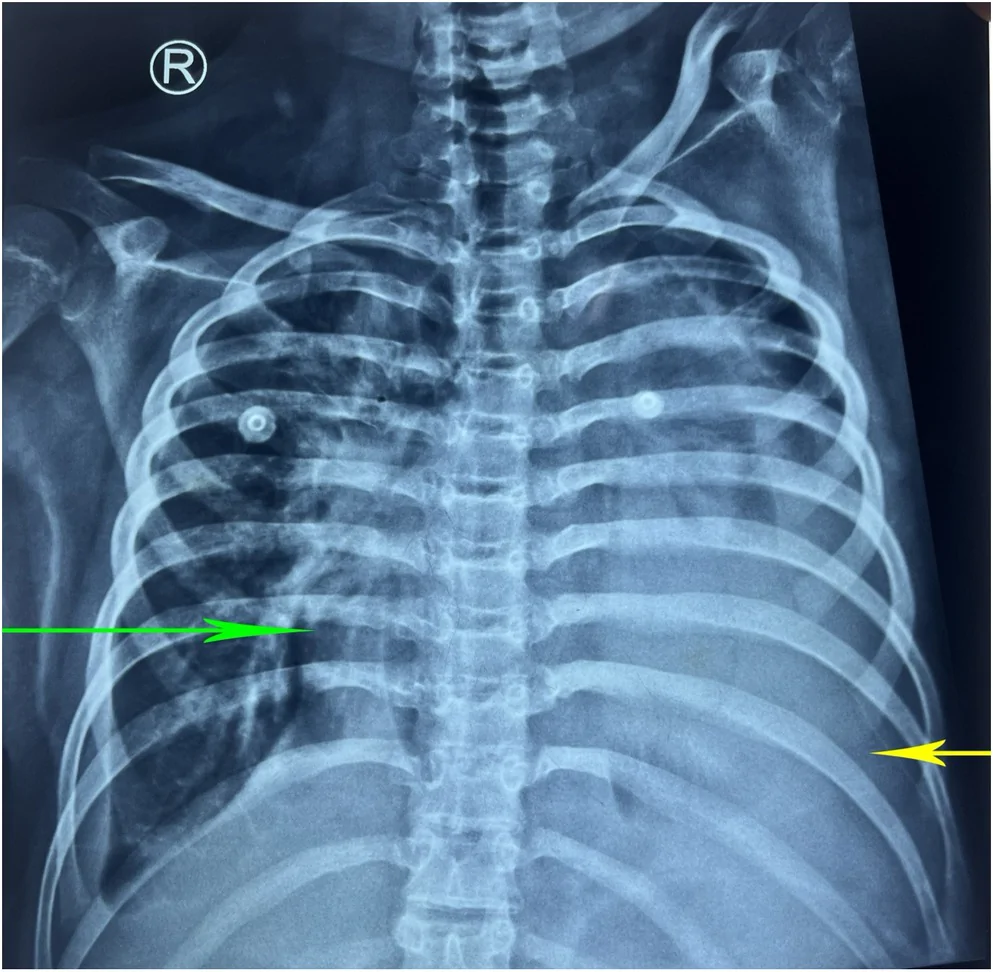
Chest radiograph on admission demonstrating a massive left‐sided pleural effusion with a small right‐sided pleural effusion, associated left lung compression, and an enlarged cardiac silhouette (yellow arrows showing pleural effusion and green arrow showing pericardial effusion).

**FIGURE 2:**
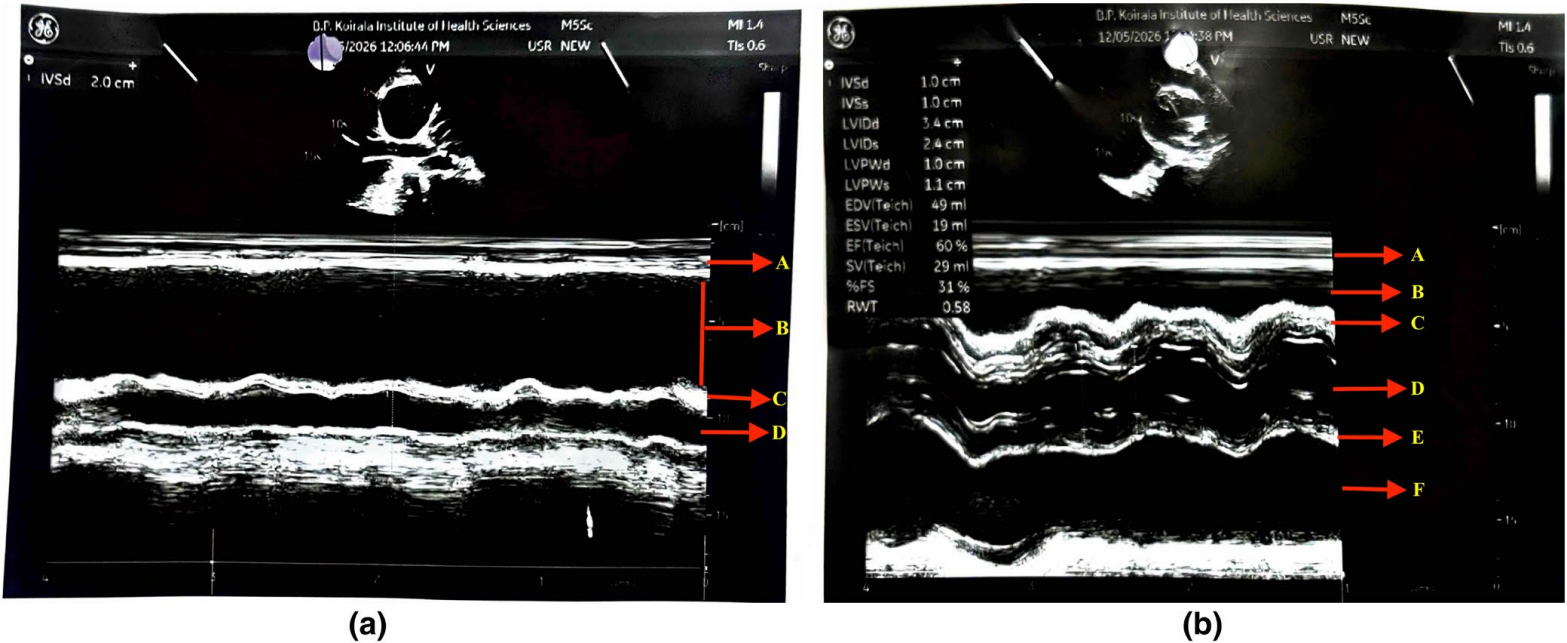
(a) Transthoracic echocardiography (subcostal view) demonstrating a massive pericardial effusion without echocardiographic evidence of cardiac tamponade. A: outer pericardium; B: pericardial fluid; C: inferior vena cava (IVC) wall; D: IVC diameter. (b) Follow‐up transthoracic echocardiography (parasternal long‐axis [PLAX] view) performed before discharge after initiation of levothyroxine replacement therapy, demonstrating marked reduction of the pericardial effusion. A: right ventricular free wall; B: right ventricular cavity; C: interventricular septum; D: left ventricular internal diameter; E: posterior wall of the left ventricle; F: pleural cavity containing fluid.

Thyroid function tests were sent in view of pericardial effusions with generalized edema. The results revealed severe hypothyroidism with low free T3 (1.02 pg/mL), low free T4 (< 0.42 ng/dL), and extremely elevated TSH (359.964 μIU/mL). Anti‐thyroid peroxidase (anti‐TPO) antibodies were markedly elevated at 1020 IU/mL supporting an autoimmune etiology most likely Hashimoto's thyroiditis. Anti‐dsDNA was 3.56 (normal < 30).

So in conjunction with the clinical presentation, autoimmune hypothyroidism due to Hashimoto's thyroiditis, and the absence of microbiological evidence of infection, the effusion was attributed to hypothyroidism. The presence of generalized edema, pleural and pericardial effusions, altered sensorium, constipation, and markedly elevated TSH suggested severe decompensated hypothyroidism with features suggestive of myxedema crisis.

## 3 Treatment

The patient was admitted to the intensive care unit (ICU) with severe hypothyroidism presenting with features suggestive of myxedema crisis, respiratory failure, and massive pleural and pericardial effusion. He was initially managed with non‐invasive ventilation (NIV) and inotropic support because of hypotension and respiratory distress.

Empirical broad spectrum antibiotic therapy was initiated for a suspected precipitating respiratory infection. Thyroid hormone replacement was started with oral levothyroxine 100 μg daily, which was subsequently increased to 125 μg daily during hospitalization. Oral levothyroxine was used because of unavailability of intravenous levothyroxine. Intravenous hydrocortisone 50 mg four times daily was administered during the acute phase.

Therapeutic pleural fluid drainage of 1 L was performed.

Serial thyroid function tests (TSH, free T3, and free T4) were monitored demonstrating gradual biochemical improvement (Table 1).

**TABLE 1:** Serial thyroid function tests.

Date	fT3	fT4	TSH
2026/05/12	1.02	< 0.42	359.964
2026/05/15	1.03	0.42	100.00
2026/05/19	1.37	0.97	90.970
2026/05/21	1.40	1.00	69.140
2026/05/24	1.56	1.01	57.151

He remained on NIV support for a total of 9 days and was subsequently weaned to Venturi mask oxygen therapy and later to room air. Hemodynamic status also improved, allowing gradual discontinuation of inotropic support.

Following clinical, biochemical, and respiratory improvement, the patient was transferred out of the ICU to the general ward for further management.

The patient was discharged from the hospital on the 16th hospital day in stable clinical condition. By the 16th day, the pericardial and pleural effusions had completely resolved. Although a limited diagnostic pericardiocentesis was performed at presentation, no therapeutic pericardiocentesis was required during the hospital course because there was no echocardiographic evidence of cardiac tamponade. The serial improvements are shown in (Figure 3).

**FIGURE 3:**
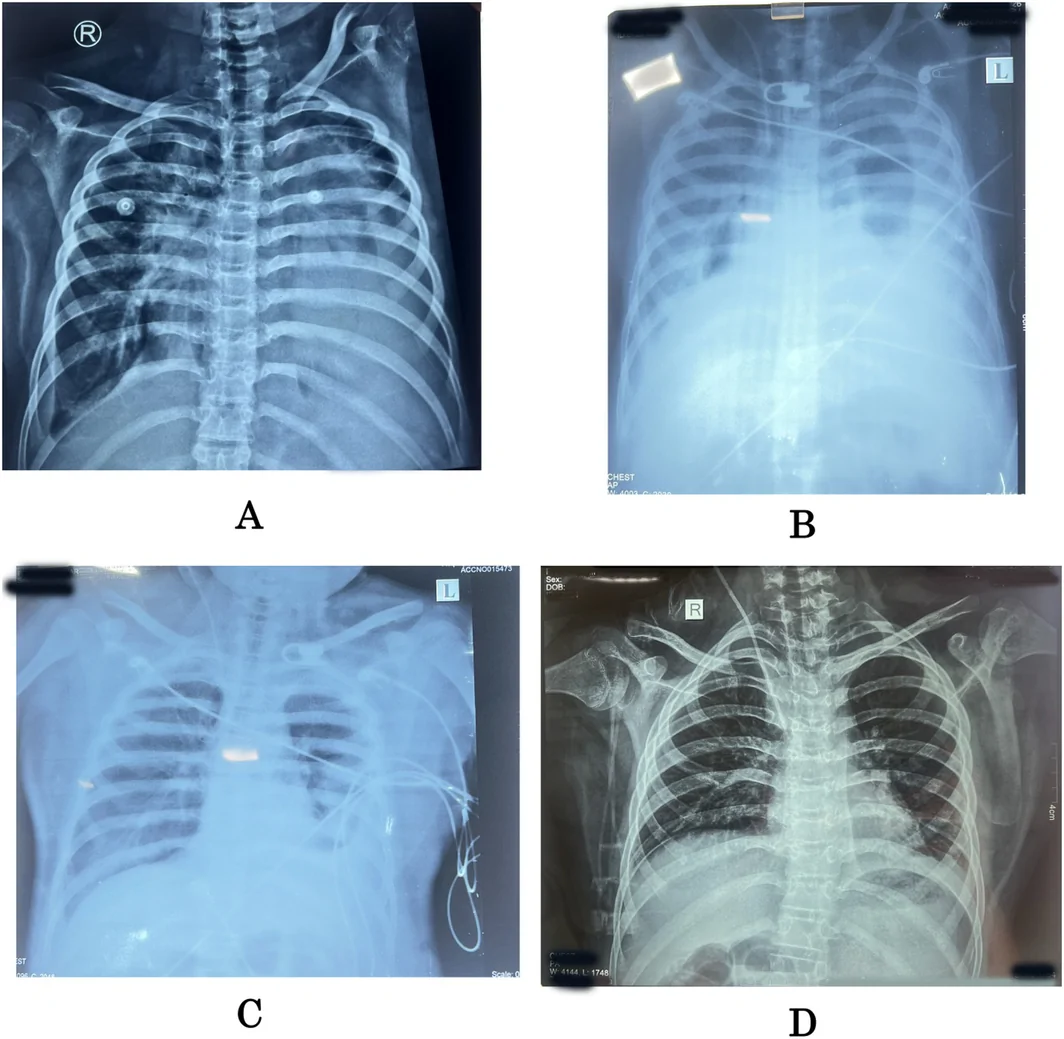
Serial chest radiographs showing progressive resolution of the massive left‐sided pleural effusion and small right‐sided pleural effusion following pleural drainage, thyroid hormone replacement, and supportive management. (A) Day 1 (admission), (B) Day 4, (C) Day 8, and (D) Day 16.

## 4 Follow‐Up

At 1‐week follow‐up, the patient remained clinically stable with no recurrence of symptoms. Repeat thyroid function testing and echocardiographic evaluation were planned at subsequent visits; however, the patient was lost to follow‐up, precluding further assessment of long‐term clinical, biochemical, and radiological outcomes.

A chronological summary of the symptom progression, diagnostic evaluation, treatment, hospitalization, discharge, and follow‐up of an adolescent with Down syndrome presenting with severe autoimmune hypothyroidism complicated by myxedema crisis, massive pericardial effusion, and bilateral pleural effusions in (Table 2).

**TABLE 2:** Timeline of clinical presentation, management, and follow‐up.

Date/Time	Clinical events
~6 weeks before admission	Progressive facial and lower‐limb swelling gradually progressed to generalized edema. Associated lethargy, irritability, reduced interaction, and intermittent constipation developed.
4–5 days before admission	Onset of shortness of breath with gradual worsening.
Day 1 (admission)	Presented with generalized edema, respiratory distress, lethargy, and altered mental status. Examination revealed hypotension, hypoxemia, distant heart sounds, and generalized non‐pitting edema.
Day 1	Chest radiography showed massive bilateral pleural effusions and cardiomegaly. Echocardiography demonstrated a massive pericardial effusion without cardiac tamponade.
Day 1	Thyroid function tests confirmed severe primary hypothyroidism (fT3 1.02 pg/mL, fT4 < 0.42 ng/dL, TSH 359.964 μIU/mL). Anti‐TPO antibodies were markedly elevated (1020 IU/mL), consistent with autoimmune hypothyroidism.
Day 2	Admitted to the intensive care unit. Treatment initiated with non‐invasive ventilation, inotropic support, empirical antibiotics, hydrocortisone, oral levothyroxine (100 μg/day), and therapeutic pleural drainage.
Days 3–9	Progressive clinical, biochemical, and respiratory improvement. Levothyroxine started at 100 μg/day then subsequently increased to 125 μg/day. Serial thyroid function tests demonstrated improving thyroid hormone levels and declining TSH.
Day 9	Successfully weaned from non‐invasive ventilation and subsequently maintained on room air.
Day 10	Transout from ICU to internal medicine ward.
Day 16 (discharge)	Marked clinical, biochemical, and radiological improvement with complete resolution of pleural and pericardial effusions. Discharged on oral levothyroxine therapy.
1‐week post‐discharge follow‐up	Clinically stable with continued symptomatic improvement and adherence to levothyroxine therapy. Repeat thyroid function testing and echocardiographic assessment were planned at subsequent follow‐up visits.
Subsequent follow‐up	Patient was lost to follow‐up; therefore, long‐term clinical, biochemical, and echocardiographic outcomes could not be assessed.

## 5 Discussion

The presence of massive pericardial effusion in our patient, along with decreased mental status, hypotension, and a precipitating illness (pneumonia), suggests long standing and severe hypothyroidism that progressed to decompensated hypothyroidism, also known as myxedema crisis. Myxedema crisis is the most severe form of long‐standing hypothyroidism and occurs when the body can no longer compensate for low thyroid hormone levels [1]. It presents with altered mental status, hypothermia, bradycardia, hypotension, hypoventilation, and hypoglycemia. It is associated with high mortality if not treated early. It is extremely rare in children and adolescents, and only a few cases have been reported [3]. Therefore, diagnosis is often delayed.

In pediatrics, diagnosis is further complicated by the lack of specific diagnostic criteria. This is particularly true in children with Down syndrome, as in our case, where many clinical features overlap. Several manifestations of hypothyroidism, including developmental delay, reduced cognitive function, decreased activity, constipation, hypotonia, and behavioral changes, may be similar to features of Down syndrome, leading to delayed diagnosis and treatment [14]. In our patient, the diagnosis was initially challenging due to overlapping features of Down syndrome, but severe hypothyroidism was eventually confirmed and showed significant improvement after thyroid hormone replacement. Despite its severity, myxedema crisis is reversible with prompt thyroid hormone therapy and treatment of the underlying trigger [2].

Hypothyroidism is common in patients with Down syndrome but the diagnosis is often delayed and missed. In a study by Fort et al. [15] thyroid dysfunction was found in about 1% (11 out of 1130 infants) with Down syndrome, compared to about 0.03% (1 in 3800) in the general newborn population. This shows a markedly higher risk in Down syndrome infants. These data highlight the importance of regular thyroid screening in patients with Down syndrome. The American Academy of Pediatrics has made guidelines for the screening of patients with Down syndrome. It recommends verification of newborn thyroid screening results, followed by TSH testing at 6 months, 12 months, and annually thereafter in children with Down syndrome [13]. This helps in prompt diagnosis of thyroid disorder in patients with Down syndrome. However, in developing countries like Nepal no such consensus guidelines are developed. As a result, patients may present at an advanced stage, as in our case, with massive pericardial effusion or myxedema crisis. To avoid this awareness among healthcare workers needs to be improved and regular thyroid screening in children and adolescents needs to be implemented.

Pericardial effusion is common in severe and unrecognized hypothyroidism. Hypothyroidism is associated with increased capillary permeability resulting in increased leakage of plasma proteins into the interstitial space and a lack of compensatory increase in lymph flow and return rate [16]. Eventually the patient may develop severe pericardial effusion often in the absence of an infectious cause. This hypothyroidism associated pericardial effusion develops slowly over weeks to months allowing the pericardium to stretch [7]. In our review of 15 reported cases (Table 3) only three patients developed cardiac tamponade. Therefore, cardiac tamponade remains an uncommon complication even in the presence of massive pericardial effusion. Rare exceptions with rapid reaccumulation of pericardial fluid and tamponade have been reported although the underlying mechanism remains unclear [6].

**TABLE 3:** Reported cases of hypothyroidism associated with pericardial effusion.

Author (year)	Case	Age	Down syndrome	TSH mIU/L	Pericardial effusion	Tamponade	Pericardiocentesis	Levothyroxine	Outcome
Yorulmaz et al. [4]		32 years	No	433	Yes	Yes	Yes	Yes	Resolution
Hallett et al. [5]		17 years	No	99.2	Yes	No	No	Yes	Resolution
Zhu et al. [3]		6 Years	No	> 150	Yes	No	No	Yes	Resolution
Anah et al. [6]		16 years	Yes	> 40	Yes	Yes	Yes	Yes	Resolution
Said et al. [7]		39 years	Yes	160	Yes	No	No	Yes	Resolution
Dinleyici et al. [8]	1	7 months	Yes	21	Yes	No	Yes	Yes	Resolution
	2	2 years	Yes	17.9	Yes	No	No	Yes	Resolution
	3	2 years	Yes	16.8	Yes	No	No	Yes	Resolution
	4	5 months	Yes	18.06	Yes	No	No	Yes	Resolution
Pinsker et al. [9]		11 years	Yes	> 800	Yes	No	No	Yes	Resolution
Bereket et al. [10]	1	17 years	Yes	> 50	Yes	No	Yes	Yes	Resolution
	2	13 years	Yes	330	Yes	No	Yes	Yes	Resolution
Werder et al. [11]	1	9 years	Yes	366	Yes	No	No	Yes	Resolution
	2	12 years	Yes	> 48	Yes	No	No	Yes	Resolution
	3	9 years	Yes	800	Yes	No	No	Yes	Resolution

Among the 15 previously reported cases (Tables 3 and 4) of hypothyroidism‐associated pericardial effusion, 12 (80%) occurred in patients with Down syndrome. Pericardial effusion was present in all cases (15/15, 100%), whereas cardiac tamponade developed in only two cases (13.33%). Therapeutic Pericardiocentesis was performed in four patients (26.66%), primarily because of cardiac tamponade or significant symptomatic effusions. All patients received levothyroxine therapy, and complete clinical resolution was reported in every case (15/15, 100%).

**TABLE 4:** Comparison of previously reported cases with the present case.

Characteristic	Previous case (*n* = 15)	Present case
Down syndrome	12 (80%)	Yes
Pericardial effusion	15 (100%)	Yes
Cardiac tamponade	2 (13.33%)	No
Diagnostic pericardiocentesis	1 (6.66%)	Diagnostic (20 mL)
Therapeutic pericardiocentesis	4 (26.66)	No
Levothyroxine therapy	15 (100%)	Yes
Outcome	Complete resolution (15/15)	Discharged with improving clinical status

Although our patient presented with hypotension requiring transient inotropic support, echocardiography demonstrated no evidence of cardiac tamponade physiology. Therefore, the hemodynamic instability was considered more likely to be multifactorial, resulting from severe hypothyroidism with features of myxedema crisis, respiratory failure, and a concomitant suspected infection rather than impaired cardiac filling secondary to the pericardial effusion itself. Given that hypothyroidism‐associated pericardial effusions typically accumulate gradually, allowing adaptation of the pericardium, and often resolve with thyroid hormone replacement, a conservative management strategy was adopted. A limited diagnostic pericardiocentesis was performed to obtain fluid for laboratory evaluation; however, therapeutic drainage was deferred because there was no echocardiographic evidence of tamponade physiology. The patient's subsequent progressive clinical, biochemical, hemodynamic, and radiological improvement following levothyroxine replacement, hydrocortisone administration, supportive care, and treatment of the suspected precipitating infection further supported this management approach. In our review of 15 reported cases (Table 3), all patients improved with levothyroxine therapy, while only five required pericardiocentesis, primarily because of cardiac tamponade or large symptomatic effusions. These findings, together with the favorable outcome observed in our patient, suggest that even massive hypothyroidism‐associated pericardial effusions can often be managed successfully with thyroid hormone replacement alone when tamponade physiology is absent. Our case therefore highlights that careful echocardiographic assessment is crucial in guiding management decisions and that invasive therapeutic pericardiocentesis may be safely reserved for selected patients with hemodynamic compromise directly attributable to cardiac tamponade.

## 6 Limitation

1. Unavailability of intravenous levothyroxine in our setting.
2. Absence of long‐term follow‐up for assessment of thyroid hormone normalization and recurrence of pericardial effusion.
3. Histopathological confirmation of autoimmune thyroiditis was not available; the diagnosis was based on clinical findings and positive anti‐TPO antibodies.

## 7 Conclusion

This case highlights that severe hypothyroidism in adolescents with Down syndrome can present as myxedema crisis and massive pericardial effusions. Because the clinical manifestations of hypothyroidism frequently overlap with those of Down syndrome, diagnosis may be delayed until life‐threatening complications develop. Thyroid function testing should be considered early in patients with Down syndrome presenting with unexplained edema, altered mental status, respiratory distress, or pericardial effusion. Recognition of this association is crucial, as even massive pericardial effusion and decompensated hypothyroidism are reversible with timely thyroid hormone replacement and supportive care, with invasive pericardiocentesis reserved for hemodynamically unstable patients or tamponade physiology only. Our patient achieved clinical, biochemical, and radiological recovery on thyroid hormone replacement and supportive care without need for therapeutic pericardiocentesis. Regular thyroid function screening in children and adolescents with Down syndrome, particularly in resource‐limited settings, remains essential for early diagnosis and prevention of severe complications.

## Author Contributions

**Bivek Mishra:** conceptualization, data curation, investigation, methodology, visualization, writing – original draft. **Pujan Pokharel:** conceptualization, methodology, software, data curation, investigation, validation, formal analysis, visualization, project administration, resources, writing – original draft, writing – review and editing. **Abinash Karki:** conceptualization, investigation, writing – review and editing, methodology, visualization, resources, project administration, supervision. **Oshin Rimal:** conceptualization, investigation, writing – review and editing. **Ayush Bakhrel:** methodology, investigation, visualization, writing – review and editing, project administration, resources, supervision. **Diwakar Koirala:** conceptualization, methodology, investigation, writing – review and editing. **Ramesh Sapkota:** conceptualization, investigation, writing – review and editing.

## Funding

The authors have nothing to report.

## Ethics Statement

Our institution does not require ethical approval for reporting individual cases or case series.

## Consent

Written informed consent was obtained from the patient for their anonymized information to be published in this article.

## Conflicts of Interest

The authors declare no conflicts of interest.

## Data Availability

The data that support the findings of this study are available from the corresponding author upon reasonable request.
